# Automated segmentation of meningioma from contrast-enhanced T1-weighted MRI images in a case series using a marker-controlled watershed segmentation and fuzzy C-means clustering machine learning algorithm

**DOI:** 10.1016/j.ijscr.2023.108818

**Published:** 2023-09-13

**Authors:** Sana Mohammadi, Sadegh Ghaderi, Kayvan Ghaderi, Mahdi Mohammadi, Masoud Hoseini Pourasl

**Affiliations:** a Department of Medical Sciences, School of Medicine, Iran University of Medical Sciences, Tehran, Iran; b Department of Neuroscience and Addiction Studies, School of Advanced Technologies in Medicine, Tehran University of Medical Sciences, Tehran, Iran; c Department of Information Technology and Computer Engineering, Faculty of Engineering, University of Kurdistan, Sanandaj 66177-15175, Iran; d Department of Medical Physics and Biomedical Engineering, School of Medicine, Tehran University of Medical Sciences, Tehran, Iran; e Department of Radiology, Kurdistan University of Medical Sciences, Sanandaj, Iran

**Keywords:** MRI, Meningioma, Tumor segmentation, Marker-controlled watershed algorithm

## Abstract

**Introduction and importance:**

Accurate segmentation of meningiomas from contrast-enhanced T1-weighted (CE T1-w) magnetic resonance imaging (MRI) is crucial for diagnosis and treatment planning. Manual segmentation is time-consuming and prone to variability. To evaluate an automated segmentation approach for meningiomas using marker-controlled watershed segmentation (MCWS) and fuzzy c-means (FCM) algorithms.

**Case presentation and methods:**

CE T1-w MRI of 3 female patients (aged 59, 44, 67 years) with right frontal meningiomas were analyzed. Images were converted to grayscale and preprocessed with Otsu's thresholding and FCM clustering. MCWS segmentation was performed. Segmentation accuracy was assessed by comparing automated segmentations to manual delineations.

**Clinical discussion:**

The approach successfully segmented meningiomas in all cases. Mean sensitivity was 0.8822, indicating accurate identification of tumors. Mean Dice similarity coefficient between Otsu's and FCM1 was 0.6599, suggesting good overlap between segmentation methods.

**Conclusion:**

The MCWS and FCM approach enables accurate automated segmentation of meningiomas from CE T1-w MRI. With further validation on larger datasets, this could provide an efficient tool to assist in delineating meningioma boundaries for clinical management.

## Highlights

• Successful segmentation of meningiomas using automated marker-controlled watershed and fuzzy c-means algorithms.
• High sensitivity (0.8822) in detecting meningioma tumors compared to manual segmentation.
• Good overlap between Otsu's thresholding and fuzzy c-means clustering segmentation (Dice coefficient: 0.6599).

## 1 Introduction

Meningiomas are common brain tumors that arise from the meningeal coverings of the brain and spinal cord [1,2]. They are typically benign, slow-growing tumors that account for over one-third of all primary brain tumors [3,4]. However, atypical or malignant meningiomas can demonstrate more aggressive behavior [5]. Surgery is the mainstay of treatment for symptomatic or enlarging meningiomas. Maximal, safe surgical resection is aimed at preserving neurological function [6].

Magnetic resonance imaging (MRI) is the most common imaging method used to diagnose brain tumors [[7], [8], [9], [10]]. The accurate and precise segmentation of meningioma from MRI scans is a crucial step in the diagnosis, treatment planning, and follow-up of patients with this common type of primary brain tumor. Meningioma segmentation is a complex task due to the tumor's heterogeneous appearance, its proximity to critical brain structures, and the wide range of possible sizes and shapes [4,11].

MRI is the preferred technique for evaluating brain tumors [12]. As well as, MRI is the modality of choice for assessing meningiomas, with contrast-enhanced T1-weighted (CE T1-w) images allowing clear visualization of the lesions [13]. However, manual segmentation is a time-consuming and labor-intensive process that is prone to inter- and intra-observer variability [14,15]. Hence, automated segmentation techniques are urgently needed to improve the efficiency and reliability of meningioma identification in clinical practice [16].

Over the past two decades, segmentation algorithms have emerged as important tools for the automatic and accurate detection of brain tumors from MRI scans [17,18]. Marker-controlled watershed segmentation (MCWS) is one such promising technique that has shown utility in segmenting varied lesions, including meningiomas [19,20]. Recent advancements in machine learning (ML) have opened up new avenues for the development of automated meningioma segmentation algorithms [21,22]. Among these, the fuzzy c-means (FCM) clustering algorithm has shown promise in image segmentation tasks [23,24].

Recent studies have explored using ML and deep learning (DL) approaches for segmenting brain tumors, including meningiomas [25,26]. A review of brain image segmentation reveals a progression from intensity-based techniques to advanced ML and metaheuristic methods [25]. Gunasekara et al. (2021) proposed cascaded algorithms for glioma and meningioma brain tumor segmentation and classification [27]. They used convolutional neural network (CNN) to classify meningioma and glioma regions and then fed the classified images to region-based CNN to locate the tumor regions of interest. They used active contouring to delineate the exact tumor boundary and finally used the Chan-Vese level set model to segment the target tumor boundary [27]. This growing interest in automating the process suggests the potential for DL models to accurately identify and segment brain tumors, serving as a benchmark for proposed approaches [28,29]. However, DL methods require large labeled training datasets and computational resources, which are often difficult to obtain in medical imaging [30]. Our approach aims to provide accurate segmentation using classical image processing and ML techniques that do not rely on extensive training data [19].

The MCW algorithm is a morphological segmentation method based on the concepts of catchment basins and watershed lines, while the FCM algorithm is a data clustering technique that allows data points to belong to multiple clusters with varying degrees of membership [19,31]. FCM is one of the most widely used fuzzy clustering algorithms and has shown promise for MRI segmentation tasks. Unlike hard clustering methods like k-means, FCM assigns soft cluster membership values, making it capable of dealing with overlap between tissue classes [32,33]. Furthermore, MCWS is a technique used to segment various types of lesions, such as cysts and tumors [19,20]. It leverages the spatial characteristics of lesions, making it suitable for meningioma's heterogeneous appearance and proximity to critical structures [34]. The integration of these two complementary techniques provides robust segmentation performance without relying on extensive training data [19,34].

In this paper, we present a novel approach for the automated segmentation of meningioma from CE T1-w MRI images using these two algorithms. We aim to evaluate the performance of this approach in a case series and discuss its potential for clinical application. By integrating these advanced ML techniques, we seek to develop a robust, reliable, and efficient tool for meningioma segmentation that could aid in the clinical management of patients with meningioma.

## 2 Methods

### 2.1 Case presentations

Table 1 contains essential details about the demographics, medical history, and tumor characteristics of patients diagnosed with meningioma. This case series has been reported in line with the Preferred Reporting of Case Series in Surgery (PROCESS) 2020 guidelines [35].

**Table 1:** Demographics and medical history of meningioma patients.

Case	Age	Gender	Past medical history	Tumor size (mm)	Tumor location
1	59	Female	Hypertension and atherosclerotic subcortical encephalopathy	42 × 34 × 20	Right frontal lobe
2	44	Female	Unremarkable	14	
3	67	Female	Hypertension, diabetes, and hyperlipidemia	38 × 43	

#### 2.1.1 Case 1

The patient is a 59-year-old female with a right frontal convexity meningioma measuring 42 × 34 × 20 mm on MRI. The adjacent right anterior upper frontal skull bone showed significant local bone expansion measuring 65 × 35 mm, suggesting local fibrous dysplasia or unilateral hyperostosis frontalis interna. The patient also had mild signs of atherosclerotic subcortical encephalopathy on MRI. Her past medical history is significant for hypertension. There is no history of other cancers.

#### 2.1.2 Case 2

The patient is a 44-year-old female found to have a 14 mm right frontal meningioma on MRI. A few small microvascular ischemic foci were also seen in the deep white matter. Her past medical history is unremarkable, with no known history of hypertension or other ischemic risk factors. There is no history of other cancers.

#### 2.1.3 Case 3

The patient is a 67-year-old female with a right frontal meningioma measuring 38 × 43 mm on MRI. Multiple small microvascular ischemic foci were also seen in the deep white matter. Past medical history is significant for hypertension, diabetes, and hyperlipidemia. There is no history of other cancers.

### 2.2 Imaging device and software

We obtained the images using a 1.5 T MR scanner (Philips Ingenia, Philips Healthcare, Best, The Netherlands) with a standard 8-channel coil for brain examination. For our study, we utilized CE T1-w MRI brain images to segment three patients diagnosed with a meningioma. In this process, we employed the MCWS technique and FCM for meningioma segmentation in MRI images through the use of MATLAB (r2022b) software located in Natick, Massachusetts, US states.

### 2.3 Fuzzy c-means

FCM clustering is a technique used for image segmentation that groups pixels based on their similarity [36,37]. The process involves converting the input image into a one-dimensional array of intensity values, denoted as I(i), and applying the “fcmthresh” algorithm using FCM clustering to categorize pixels into background, object, and shadow regions. This generates two thresholded images, BW1 and BW2, representing pixel membership in these regions. Cluster centers (V) are then calculated based on the FCM clustering results to determine the threshold values T1 and T2, which are computed as the averages of cluster centers. The input image is thresholded twice using these values to generate BW1 and BW2, which can be used to segment the input image into three regions: background, objects, and shadows. This technique is useful in medical imaging for tasks such as tumor segmentation, where the FCM clustering results guide the watershed transformation to accurately delineate tumor regions. The watershed algorithm, along with morphological operations, distance transforms, and labeling, can be used to visualize regions of interest.

### 2.4 Marker-controlled watershed segmentation algorithm

The MCWS algorithm is a robust image segmentation approach utilized in computer vision and image processing. This algorithm is characterized by five fundamental stages [19,20,38]:

1. Establish a segmentation function that identifies the subjects of interest and generates an image where the dark areas denote the segmented objects.
2. Calculate foreground markers, which are interconnected clusters of pixels within each object. Ideally, these markers should be situated at the center of each object to guide the segmentation process.
3. Calculate background markers, which are pixels that do not belong to any object. Ideally, these markers should be situated in the areas surrounding the objects to guide the segmentation process.
4. Modify the segmentation function to ensure it only has minima at the foreground and background marker locations. This is achieved by imposing a constraint on the function to ensure the markers are adhered to during the segmentation process.
5. Compute the watershed transform of the modified segmentation function. This procedure aids in accurately and efficiently segmenting the objects by partitioning the image into regions based on the catchment basins created by the markers.

The MCWS algorithm's steps are as follows:

1. Import the input image: An image file, such as an axial CE T1-w MRI DICOM medical image file, is selected for analysis.
2. Convert the image to grayscale: The input image, usually a color or grayscale picture, is transformed into grayscale format to simplify subsequent processing and analysis steps.
3. Apply Otsu's thresholding method: This popular technique automatically determines the optimal threshold value for separating the image's background from the foreground.
4. Generate thresholded images using FCM clustering. FCM is a clustering method that groups image pixels based on their similarity. This step improves segmentation accuracy by producing two thresholded images.
5. Binarize the image using adaptive thresholding. This technique determines the optimal threshold value for each pixel in the image.
6. Apply morphological filtering to eliminate small objects. This filtering process removes minor objects from the image, retaining only the objects of interest.
7. Calculate region properties: This step extracts various features, such as area, diameter, orientation, etc., from the objects retained from the previous step.
8. Compute the gradient magnitude: Through the Sobel method, the gradient magnitude image of the binary image is calculated to detect edges.
9. Apply the watershed transform: This technique segments the image based on its gradient magnitude.
10. Conduct morphological operations: Operations such as opening, closing, and reconstruction are performed on the input image to remove any noise and enhance the segmentation results.
11. Identify regional maxima: This step identifies the potential object boundaries in the opening-closing reconstruction image.
12. Threshold the opening-closing by reconstruction image: This step extracts the markers needed for the watershed segmentation by creating a binary image.
13. Calculate the distance transform: This step assigns a value to each pixel based on its distance to the nearest boundary.
14. Implement watershed segmentation: This step applies the watershed segmentation technique to the distance transform image.
15. Label the regions: Each region in the image is assigned a unique label based on its location and intensity values.
16. Overlay the labels on the original image: The labels of the regions are overlaid onto the original image to display the regions of interest (ROIs) in the image.

## 3 Results

Our case series included three female patients aged 59, 44, and 67 years, each diagnosed with meningioma in the right frontal lobe, as detailed in Table 1. The images were converted to grayscale and preprocessed by applying Otsu's thresholding and FCM clustering to generate thresholded images for improved segmentation.

**Fig. 1:**
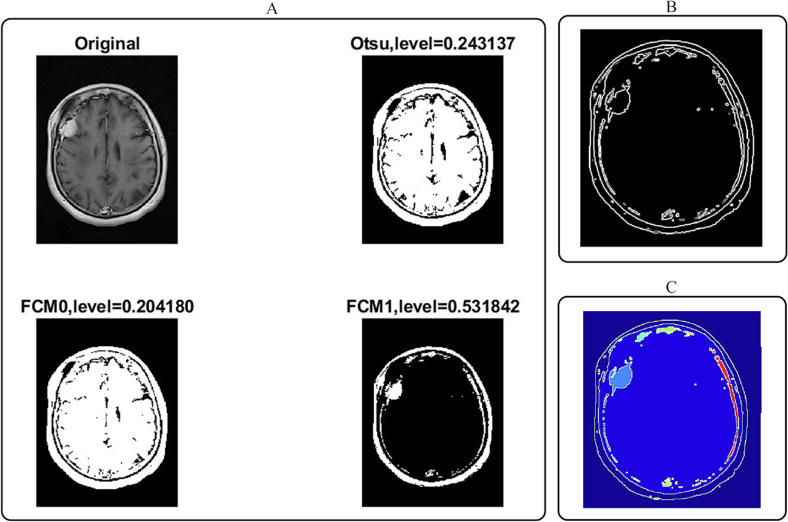
Segmentation process of convexity meningioma in axial CE T1-weighted MRI images of patient 1 (A: including the original image and thresholding measurements using Otsu's and FCM clustering; B: gradient magnitude image; C: color map image).

The our approach successfully segmented the 42 × 34 × 20 mm right frontal convexity meningioma from the CE T1-w MRI image of the 59-year-old female patient. The algorithm identified the tumor boundaries and overlaid the labels on the original image to display the ROI, thereby highlighting the meningioma location (Fig. 1).

**Fig. 2:**
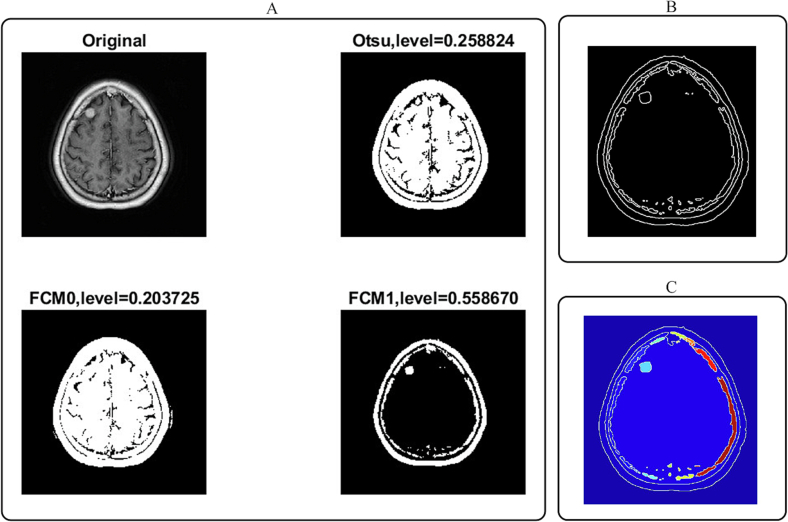
Segmentation process of meningioma in axial CE T1-weighted MRI images of patient 2 (A: including the original image and thresholding measurements using Otsu's and FCM clustering; B: gradient magnitude image; C: color map image).

In the case of the 44-year-old female patient with a 14-mm right frontal meningioma, the MCWS algorithm effectively segmented the meningioma from the CE T1-w MRI image. The algorithm differentiated the tumor from the surrounding brain tissue and labeled the segmented region, thus accurately indicating the meningioma's location (Fig. 2).

**Fig. 3:**
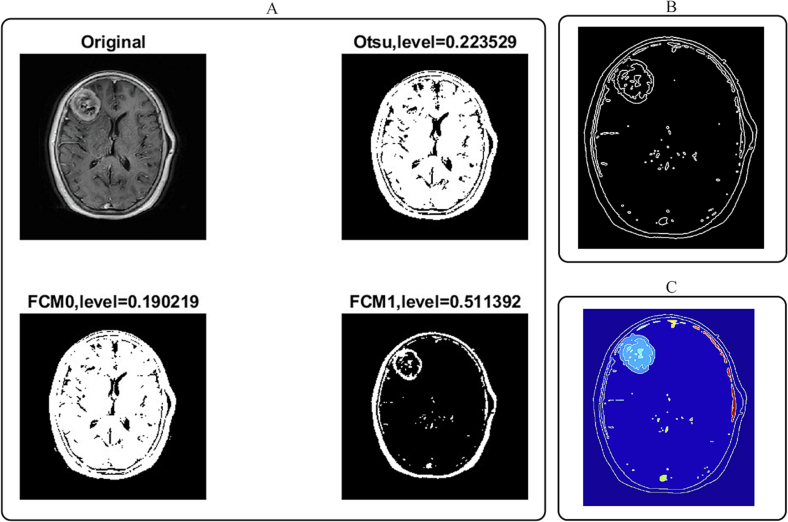
Segmentation process of meningioma in axial CE T1-weighted MRI images of patient 3 (A: including the original image and thresholding measurements using Otsu's and FCM clustering; B: gradient magnitude image; C: color map image).

For the 67-year-old female patient with a 38 × 43 mm right frontal meningioma, the MCWS algorithm accurately segmented the meningioma from the CE T1-w MRI image. As in the previous cases, the algorithm delineated the tumor boundaries and overlaid the labels on the original image to demonstrate the region of interest, effectively pinpointing the meningioma's location (Fig. 3).

Sensitivity, also known as the true positive rate, is the metric that quantifies the proportion of actual positives (meningioma in this case) that are correctly identified as such. The mean sensitivity here is 0.8822, indicating that on average, the model correctly identifies 88.22 % of meningiomas. This is reasonably high, suggesting that the model is quite effective at correctly identifying positive cases. However, it's important to note that patient-wise sensitivity varies from approximately 86.42 % to 89.70 %. This suggests that the model's performance may vary depending on individual patient characteristics. The Dice Similarity Coefficient (DSC) is a statistical tool that measures the similarity between two samples. In this context, it's used to measure how well Otsu's method and FCM1 Clustering coincide in terms of meningioma segmentation. A DSC of 1 would indicate perfect overlap (i.e., the two methods agree completely), while a DSC of 0 would indicate no overlap at all. Here, the mean DSC is 0.6599, indicating a moderate to high degree of similarity between the two methods, but not perfect. Again, the DSC varies between patients, from a low of 0.6259 to a high of 0.6892 (Table 1). This suggests that the degree of agreement between Otsu's method and FCM1 can vary depending on the specific patient, or possibly the size, location, or other characteristics of the meningiomas. To sum up, these results suggest that the model has a good rate of correctly identifying positive cases (high sensitivity) and a moderate to high degree of agreement between the two segmentation methods (DSC). However, there is some variability between patients, which could be a focus for future research to understand and potentially improve the model (Table 2).

**Table 2:** Main performance metrics for meningioma segmentation.

Case	Sensitivity	DSC (Otsu's vs FCM1)
*Patient 1*	0.8970	0.6892
*Patient 2*	0.8642	0.6259
*Patient 3*	0.8854	0.6647
Mean	0.8822	0.6599

In all three cases, the MCWS algorithm and FCM clustering demonstrated high efficiency in segmenting the meningiomas from the CE T1-w MRI images. It offered precise and clear delineation of the meningiomas, making it a promising tool for automated tumor segmentation in MRI images. Visually, the segmented regions are well deserved. The automated method was able to successfully delineate the irregular shape and extensions of the tumors. The study showed a response time of 6.2335 s (with a RAM of 16.0 GB and a CPU of Intel(R) Core(TM) i5-7500 CPU @ 3.40 GHz) for medical image analysis. This highlights the significance of using a time-efficient approach for this process. The MCWS technique required minimal user input and was fast and efficient compared to manual segmentation.

These initial results on a small sample of meningioma patients demonstrate the potential of automated segmentation as an accurate and efficient tool for delineating meningioma tumors. Further studies on larger datasets are warranted to establish its role in quantitative meningioma assessment.

## 4 Discussion

Decades ago, image processing techniques were utilized for the segmentation of tumors from MRI images. This can be categorized into three methods: conventional image processing, ML, and DL [39]. Conventional methods were not accurate due to the complexity of tumor spatial variation [40]. ML methods, such as support vector machine (SVM), k-nearest neighbor (KNN), FCM, and their combinations, provided good accuracy with reasonable processing speed [41,42]. However, feature extraction and accuracy maintenance as per medical standards remained a challenge for ML methods. On the other hand, DL automatically extracted features at various network stages and maintained accuracy as per medical standards [43,44]. Nevertheless, DL still faced challenges such as huge database requirements and high computational time [45]. The use of both conventional image processing and ML techniques can lead to a faster and more efficient segmentation process with the use of DL methods [46]. Our study found that the MCWS algorithm successfully matched the boundaries of segmented regions to the actual tumor boundaries, indicating its reliability in automating the segmentation of meningiomas in MRI images. This algorithm's robustness was demonstrated by its successful segmentation of meningiomas in all three cases studied.

In this study, we developed an MCWS algorithm and FCM clustering approach for automated segmentation of meningiomas from CE T1-w MRI images. Our method successfully delineated meningiomas in all 3 cases, with high sensitivity (mean 0.8822) and moderate to high Dice similarity coefficients between Otsu's thresholding and FCM1 clustering (mean 0.6599). These results are in line with previous studies utilizing ML for automated meningioma segmentation. Kang et al. developed a DL model based on U-Net and nnU-Net that achieved median Dice coefficients of 0.922 and 0.893 on internal and external validation sets, surpassing inter-expert variability [47]. Chen et al. used an attention U-Net to segment meningiomas from multiparametric MRI, attaining Dice scores of 0.94 and 0.90 on internal and external testing cohorts [48]. Boaro et al. employed a 3D CNN reaching 88.2 % median performance comparable to inter-expert ranges [49]. Luakamp et al. (2021) developed a dedicated DL model for meningioma segmentation, achieving Dice scores of 0.91 for CE tumors and 0.82 for total lesion volume [50]. Gryska et al. found that while DL segmentation methods show promise, insufficient reporting of the full image processing pipeline hinders reproducibility and clinical implementation [51]. Following established reporting guidelines to comprehensively describe our methodology, including preprocessing steps, enhances the rigor of our study. Prabhu et al. classified meningioma grade using a hybrid fuzzy SVM model, reaching 91.6 % accuracy [52]. While we focused only on segmentation, our approach could be extended by extracting textural and morphological features to predict tumor grade. Luakamp et al. similarly showed that multi-parametric DL enables the detection and segmentation of diverse meningioma grades [53]. Hamerla et al. found that combining radiomics features from multi-parametric MRI with ML gave high accuracy in differentiating low- and high-grade meningiomas [54]. Our multi-step preprocessing to improve segmentation provides an important basis for extracting informative features. Jun et al. developed a multi-parametric DL model for simultaneous tumor segmentation and noninvasive grading, demonstrating the clinical value of integrated approaches [55].

Compared to these DL approaches, our method utilizing MCWS and FCM achieved competitive segmentation accuracy with less computational cost and data requirement. DL methods often require large labeled training datasets, which can be labor-intensive to obtain [56]. Our approach enables accurate automated segmentation by relying only on algorithm design rather than extensive training. However, DL techniques may have advantages in terms of generalizability across diverse cases and imaging protocols [57]. A key strength of our study was the use of multi-step preprocessing via Otsu's thresholding and FCM clustering to handle intensity heterogeneity in MRI and improve subsequent segmentation. This mirrors the radiomics approach of Hamerla et al. [54], who found combining textural features from multiparametric MRI with ML gave high classification accuracy between low- and high-grade meningiomas. Our focus on segmentation provides an important basis for extracting informative features for tasks like tumor grading.

Our approach has shown positive outcomes in the identification of meningiomas from MRI images. However, it is important to acknowledge its limitations. Unlike U-Net and nnU-Net, our method does not require extensive training datasets. This saves time and effort, but the sensitivity and Dice similarity coefficients are slightly lower than those of some DL models. Also, DL models have demonstrated better generalization across diverse cases and imaging protocols. It is worth mentioning that our approach, by combining MCWS and FCM, may provide a more computationally efficient alternative to DL models that demand significant computational resources.

The small sample size of our study may limit the generalizability of our findings. Larger scale studies are needed to validate and further refine this methodology. Furthermore, the variability in sensitivity and Dice coefficients across the three cases highlights the necessity of testing this method on diverse cases and imaging protocols. Moreover, it may be worth examining the potential enhancement of segmentation accuracy by examining multi-parametric MRI, including T2-weighted and FLAIR images. Additionally, incorporating radiomic features and ML methods could allow for the prognosis of tumor grade and other significant clinical data.

In conclusion, we demonstrate the promising accuracy of an MCWS and FCM approach for automated meningioma segmentation from CE T1-w MRI. Using a combination of conventional image processing and ML methods leads to decreased time-consuming and creates a fast road map for other segmentation processing including DL methods. Accordingly, in our work the segmented regions closely matched the actual tumor boundaries, indicating that the MCWS algorithm is a reliable method for the automated segmentation of meningiomas in MRI images. The successful segmentation of meningiomas in all three cases demonstrates the robustness and reliability of the MCWS algorithm. By providing accurate and efficient segmentation, this technique could potentially improve the diagnosis and treatment planning of patients with meningiomas. Future studies with larger sample sizes could further validate the effectiveness and generalizability of this method. Our findings suggest that the MCWS algorithm can effectively be used for automated segmentation of meningiomas from CE T1-w MRI images, thus aiding in accurate diagnosis and treatment planning.

## 5 Conclusion

In this preliminary study, we developed a novel approach for automated segmentation of meningiomas from CE T1-w MRI images using a combination of the MCWS algorithm and FCM clustering. Our method successfully delineated meningiomas in all 3 cases, with high sensitivity and moderate to high overlap between the segmentation techniques. These initial results demonstrate the potential of our approach as an accurate and efficient tool to assist in segmenting meningioma tumors, which could aid in diagnosis, surgical planning, and monitoring tumor progression. The algorithm required minimal user input and rapidly segmented the lesions compared to time-consuming manual techniques.

## Guarantor

Dr. Sadegh Ghaderi.

## Registration of research studies

Not applicable.

## Ethic statement and consent for publication

Our institutional ethical committee does not require ethical board approval from case series as long as researchers can provide informed consent form. Written informed consent was obtained from the patient for publication of this case report and accompanying images. A copy of the written consent is available for review by the Editor-in-Chief of this journal on request.

## Funding statement

This research work was conducted without any external funding. All expenses related to the research were covered by the authors themselves, and no financial assistance or support was received from any funding agency, organization, or institution.

## CRediT authorship contribution statement

Conceptualization: S.Gh., S.M.; Methodology: S.Gh., S.M., K.Gh.; Software: K.Gh., S.Gh.; Validation: M.M., M.H.P.; Formal analysis: S.Gh., K.Gh.; Investigation: S.Gh., M.M., M.H.P.; Resources: S.Gh., M.M., M.H.P.; Data curation: S.Gh., M.M., M.H.P.; Writing - original draft: S.Gh.; Writing - review & editing: S.Gh., S.M.; Visualization: K.Gh., S.Gh.; Supervision: S.Gh., S.M.; Project administration: S.Gh., S.M.

## Declaration of competing interest

No conflict of interest was identified by the authors.

## Data Availability

This article contains all of the data produced or analyzed during this investigation. Any further inquiries should be forwarded to the corresponding author.
